# Aldehyde dehydrogenase 2 polymorphism is associated with chemotherapy‐related cognitive impairment in patients with breast cancer who receive chemotherapy

**DOI:** 10.1002/cam4.5319

**Published:** 2022-10-06

**Authors:** Senbang Yao, Wen Li, Shaochun Liu, Yinlian Cai, Qianqian Zhang, Lingxue Tang, Sheng Yu, Yanyan Jing, Xiangxiang Yin, Huaidong Cheng

**Affiliations:** ^1^ Department of Oncology, The Second Affiliated Hospital of Anhui Medical University Anhui Medical University Hefei Anhui China; ^2^ Cancer and Cognition Laboratory Anhui Medical University Hefei Anhui China

**Keywords:** breast cancer survivors, chemotherapy, cognitive impairment, CRCI

## Abstract

**Background:**

Chemotherapy‐related cognitive impairment (CRCI) is a common but easily overlooked condition that markedly affects the quality of life (QOL) of patients with breast cancer. The rs671 is a common gene polymorphism of aldehyde dehydrogenase 2 (*ALDH2*) in Asia that is involved in aldehyde metabolism and may be closely related to CRCI. However, no study has yet summarised the association between *ALDH2* and CRCI.

**Methods:**

This study enrolled one hundred and twenty‐four patients diagnosed with breast cancer according to the pathology results, genotyped for *ALDH2* single‐nucleotide polymorphisms (SNP) to explore these. The mini‐mental state exam (MMSE), verbal fluency test (VFT), and digit span test (DST) results were compared in these patients before and after chemotherapy (CT).

**Results:**

We found that patients with *ALDH2* gene genotypes of rs671_GG, rs886205_GG, rs4648328_CC, and rs4767944_TT polymorphisms were more likely to suffer from cognitive impairment during chemotherapy. A trend toward statistical significance was observed for rs671_GG of DST (z = 2.769, *p* = 0.006), VFT (*t* = 4.624, P<0.001); rs886205_GG of DST (z = 3.663, P<0.001); rs4648328_CC of DST (z = 2.850, *p* = 0.004), VFT (*t* = 3.477, *p* = 0.001); and rs4767944_TT of DST (z = 2.967, *p* = 0.003), VFT (*t* = 2.776, *p* = 0.008). The cognitive indicators of these patients significantly decreased after chemotherapy (*p* < 0.05). The difference in *ALDH2* rs671 was most obvious.

**Conclusion:**

Our results showed what kinds of *ALDH2* genotyped patients that are more likely to develop CRCI. In the future, it may be possible to infer the risk of CRCI by detecting the single‐nucleotide locus of *ALDH2* that is conducive to strengthening clinical interventions for these patients and improving their QOL. More importantly, this study has important implications for Asian women with breast cancer as *ALDH2* rs671 is a common polymorphism in Asians.

## Graphical abstract

It may be possible to infer the risk of CRCI by detecting the single‐nucleotide locus of ALDH2 in patients with breast cancer who receive chemotherapy, which is conducive to strengthening clinical interventions for these patients and improving their QOL.

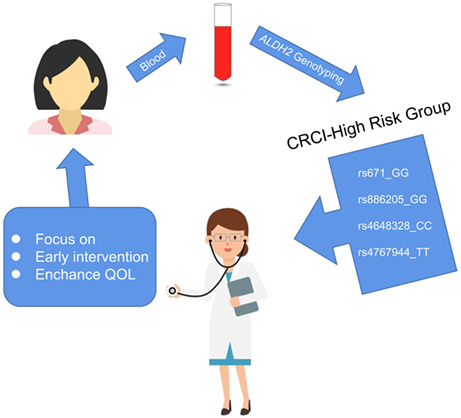

## 1 INTRODUCTION

Breast cancer is the most common type of malignant tumour in women worldwide,^1^ representing nearly 25% of all cases^2^ and accounting for 6.5% of mortalities globally.^3^ In China, the incidence of breast cancer is 7.33%, which ranks first among malignant tumours in females, with a death rate of approximately 6.9%.^4^, ^5^, ^6^ Patients with breast cancer often need multiple chemotherapy treatments.^7^, ^8^ The physical health of patients with breast cancer is severely affected by the somatic side effects of chemotherapy.^9^ However, a cognitive decline caused by chemotherapy also affects these patients' quality of life (QOL). Chemotherapy‐related cognitive impairment (CRCI) is used to define chemotherapy‐induced cognitive impairment in patients with cancer.^10^ Currently, many countries and scientific research institutions are committed to exploring the neurobiological mechanisms of CRCI.^11^, ^12^ Further study is required on how to predict which patients' cognitive function is more likely to be affected by chemotherapy. Functional MRI is effective for exploring patient cognition; however, it is expensive and not widely available.

Studies have found that the effects of chemotherapy on cognition may be due to the accumulation of metabolites such as aldehyde, tau protein,^13^ conjugated dienes,^14^ hydroperoxides,^15^, ^16^ and so on. Among these, the aldehyde is more important in inducing oxidative stress and neuroinflammation. Because oxidative stress is one important mechanism for CRCI,^17^ the normal metabolism of aldehyde can reduce neuroinflammation responses and maintain normal cognitive function. The protein encoded by the *ALDH2* (aldehyde dehydrogenase 2) gene is a key enzyme in toxic aldehyde metabolism that converts aldehyde into acid.^18^ Therefore, *ALDH2* may be involved in influencing the extent of cognitive impairment in patients with breast cancer who receive chemotherapy. The *ALDH2* single‐nucleotide polymorphisms (SNP) in rs671 is significant in Asian populations as 70% of these patients possess *ALDH2* wild‐type (GG, rs671), while 30% have *ALDH2* variants (G/A or A/A, rs671). Those with *ALDH2* variants have greatly reduced ability to metabolise aldehydes and are more susceptible to cognitive impairment.^19^, ^20^ Therfore, *ALDH2* has received extensive attention in cancer and cardiovascular disease.^21^, ^22^ The cognitive regulatory function of *ALDH2* in Alzheimer's disease (AD),^23^ Parkinson's disease,^24^ and diabetes‐associated cognitive impairments^25^ has received extensive attention. Yu et al. found that the *ALDH2* rs671 genotype was correlated with cognitive status in patients with Parkinson's disease.^24^ Ying et al. found that *ALDH2* upregulation can significantly improve cognitive function in AD mice.^23^ A Stanford University study found that low *ALDH2* activity could contribute to increased cytotoxicity by producing more reactive oxygen species (ROS) during chemotherapy,^26^ and ROS is an important factor affecting cognitive function.^27^ Therefore, it is worth exploring whether *ALDH2* plays a similar role in CRCI.

Previous studies on the genetic risk of CRCI have focused on the catechol‐O‐methyltransferase (*COMT*) gene. The *COMT* (rs165599) polymorphism has been reported to contribute to CRCI in patients with breast cancer.^28^ Recent studies have also found that three SNPs in genes related to cognitive function (*APOE* rs429358, *ANKK1* rs1800497, and *TOMM40* rs10119) are closely associated with CRCI.^29^ As *ALDH2* rs671 is an important site that determines its functional activity, it is of great significance to explore the activity of its related sites.

The current study confirmed that *ALDH2*'s role in cognitive function is important. The *ALDH2* genotype may be associated with cognitive function after chemotherapy. Currently, no studies have explored this topic. This study was conducted to determine whether *ALDH2* genotyping can identify which groups are more likely to be affected by cognitive function during chemotherapy. Positive results will have important implications for improving their QOL.

## 2 MATERIALS AND METHODS

### 2.1 Patients

This study enrolled one hundred and twenty‐four patients diagnosed with breast cancer according to pathology results. The histopathological type of breast cancer was determined on the basis of the WHO classification criteria of breast tumours. The tumour specimens were histologically graded depending on the Nottingham grading system. IHC staining was performed in all the cases for ER, PR, HER‐2, and Ki‐67 biomarkers for molecular pathology examination. The corresponding reference guide containing the WHO classification of breast tumours^30^ are the AJCC TNM staging system for breast cancer,^31^ the NCCN Guidelines for Breast Cancer,^32^ and St. Gallen International Expert Consensus.^33^ The patient cohort consisted of 124 females with an average age of 49.5 ± 10.50 years (mean and standard deviation). The baseline clinical and demographic characteristics of enrolled patients are presented in Table 1. The patients were recruited at the Second Affiliated Hospital of Anhui Medical University between September 2017 and August 2021. The exclusion criteria were as follows:(1) received radiotherapy and other treatments in the past; (2) had brain metastasis or cachexia; (3) suffered from bipolar disorders such as depression and anxiety; and (4) had impaired cognition as a result of other diseases, such as Alzheimer's disease, vascular dementia, and cerebral trauma. All patients provided informed consent before participating in this study.

**TABLE 1:** Clinical and demographic characteristics of the research groups

	N/Mean ± SD	Percentage
Total number of patients	124	100%
Age (years)	49.5 ± 10.50	/
Education (years)	10.3 ± 3.86	/
Molecular classification		
Luminal A	23	18.55%
Luminal B	47	37.90%
HER‐2 overexpression	34	27.42%
TNBC	20	16.13%
Pathological type		
Invasive Carcinoma No Special Type	115	92.74%
Invasive Carcinoma Special Type	1	0.81%
Noninvasive carcinoma	7	5.65%
Other type	1	0.81%
KPS		
100	2	1.61%
90	71	57.26%
80	33	26.61%
70	18	14.52%

Abbreviations: KPS, Karnofsky performance status; TNBC, triple‐negative breast cancer.

### 2.2 Genotyping

The collected peripheral venous blood (3–5 ml) was stored in an ethylenediamine tetraacetic acid dipotassium salt (EDTA‐K2) tube at −80°C. A genomic QIAGENE kit (Cat#69504, Shanghai Genesky Bio‐Tech Co, Ltd) was used to extract genomic DNA from the blood. The DNA samples were kept at −20°C. An improved multiplex ligase detection reaction (iMLDR) was used for genotyping. Different fluorescently labelled allele‐specific oligonucleotide probe pairs were used to distinguish the alleles of each SNP. Different SNPs were recognised by distinct extended lengths at the 3'end. Two negative controls were used; one was a double‐distilled water template and the other was a DNA sample without primers, with other conditions remaining the same. Repeated tests were conducted, and the results were consistent. Finally, 5% of the total DNA samples were randomly selected and repeated for iMLDR genotyping using an ABI3730XL automated sequencer (Applied Biosystems), and the results were consistent to confirm the iMLDR results. The sequencing results were uploaded as supplementary raw data which can be opened with GeneMapper 4.1 (AppliedBiosystems, USA) software. The picture of the iMLDR SNP genotyping schematic diagram was uploaded in the manuscript as Figure S1. The picture of iMLDR (Sample 110) was uploaded in the manuscript as Figure S2, and other samples can also be opened with GeneMapper.

### 2.3 Cognitive screening test

The mini‐mental state exam (MMSE) was used to assess cognizant ability, including spatial and time recognition, calculation, short‐term memory, language, and visual–spatial abilities. It is mainly used to quickly check whether the subject has intellectual disabilities such as mental retardation and dementia. MMSE is one of the most influential cognitive screening tools. It has been widely used in families and communities because of its simple and convenient characteristics.

### 2.4 Neuropsychological tests

Programmatic neuropsychological tests were performed after starting chemotherapy and after six cycles of postoperative adjuvant chemotherapy to evaluate memory and cognitive function. The verbal fluency test (VFT) was used, in which patients were required to say as many animal names as possible within 60 s. The digit span test (DST) was used to evaluate short‐term memory, in which patients were asked to repeat a random sequence of numbers. The number of digits repeated in the correct order determined the score.

### 2.5 The expression of ALDH2 in brain

The Human Protein Atlas (HPA) has been used to explore the expression of *ALDH2* in the brain.^34^ HPA was launched in 2003 as a project initiated by the Swedish scientific research sector to explore and summarise the expression of all human proteins in tissues and organs using ‐omics techniques (https://www.proteinatlas.org). The expression of *ALDH2* in the brain was explored using the BRAIN section of the HPA. *ALDH2* was also analysed in hippocampal formation and amygdala.

### 2.6 Statistical analysis

The proportions of qualitative variables were calculated. Means and standard deviations (SD) of quantitative variables were calculated. The data were tested for normality. The paired t‐test and paired Wilcoxon signed‐rank test were performed for normal and non‐normal distributions in continuous variable data, respectively. The independent‐samples t‐test and Mann–Whitney U‐test were performed for normal and non‐normal distribution in continuous variable data, respectively. Differences were considered statistically significant when the probability value was <0.05. The mean and SD of cognitive outcomes from similar studies and our previous pre‐experimental tests were used for sample size estimation.^24^, ^35^, ^36^, ^37^, ^38^, ^39^, ^40^, ^41^ With the test level of 0.05 on both sides and 80% power (1–β), the sample size was estimated using PASS (Power Analysis and Sample Size Software, version 15). GraphPad Prism 5.0, R language, and SPSS software (version 21.0) were used for statistical analysis and visualisation.

## 3 RESULTS

### 3.1 Process and enrolled patient profiles of the research

**FIGURE 1:**
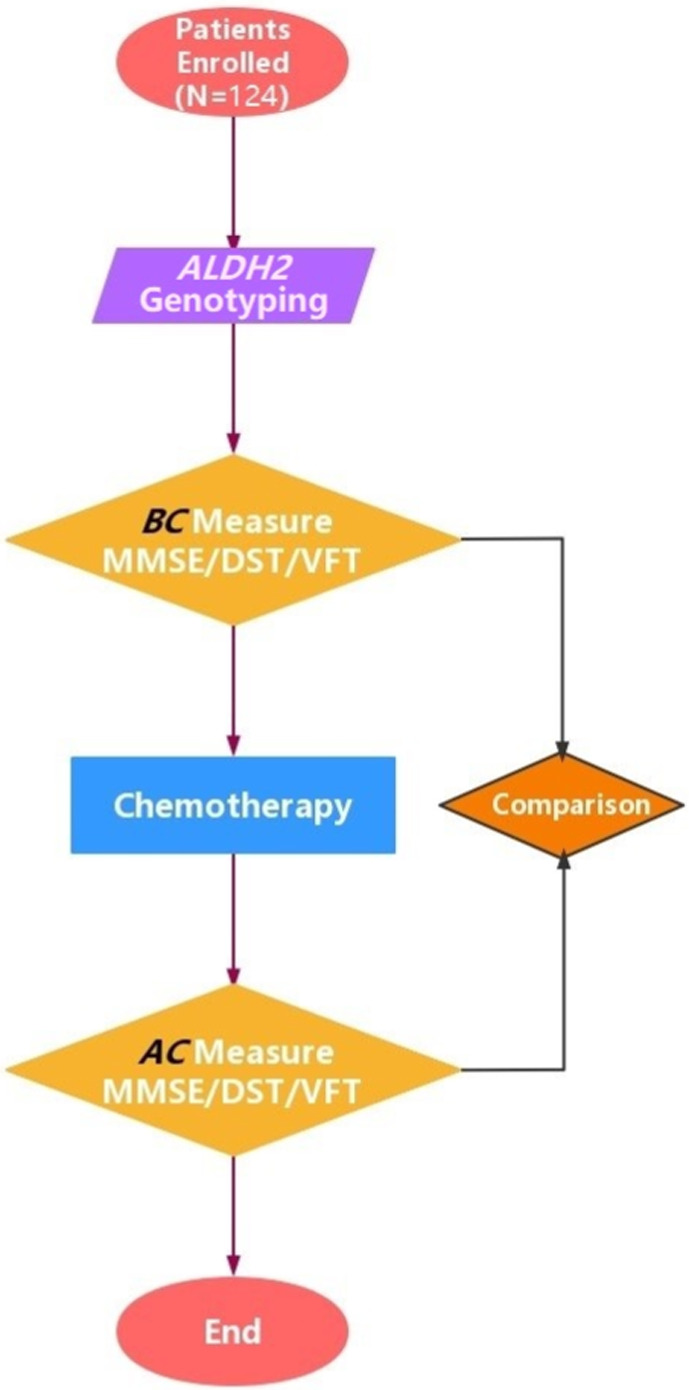
Technical process of this study

A flow diagram of our research strategy and the analytical method is shown in Figure 1. The baseline clinical and demographic characteristics of the 124 enrolled patients are presented in Table 1. General information about the four genotyped SNPs loci of the *ALDH2* gene is shown in Table 2.

**TABLE 2:** General information about four genotyped SNP loci of the *ALDH2* gene

SNP	*ALDH2*			
	rs671	rs886205	rs4648328	rs4767944
CHR	12	12	12	12
Allele position	111,803,962	111,766,623	111,784,984	111,771,537
Ref allele	G	A	C	C
Alt allele	A	G	T	T
MAF (patients in this study)	0.214	0.102	0.278	0.380
MAF (1000 g‐CHBS)	0.216	0.132	0.272	0.406
*P* (HWE test)	1.000	1.000	0.103	0.840

Abbreviations: 1000 g‐CHBS; 1000 Genomes of Han Chinese, Alt allele, alternative allele; CHR, chromosome; HWE, Hardy–Weinberg equilibrium; MAF, minor allele frequency; Ref allele; reference allele; SNP, single‐nucleotide polymorphisms.

### 3.2 Cognitive abilities of *ALDH2*_rs671 patients before and after chemotherapy

**FIGURE 2:**
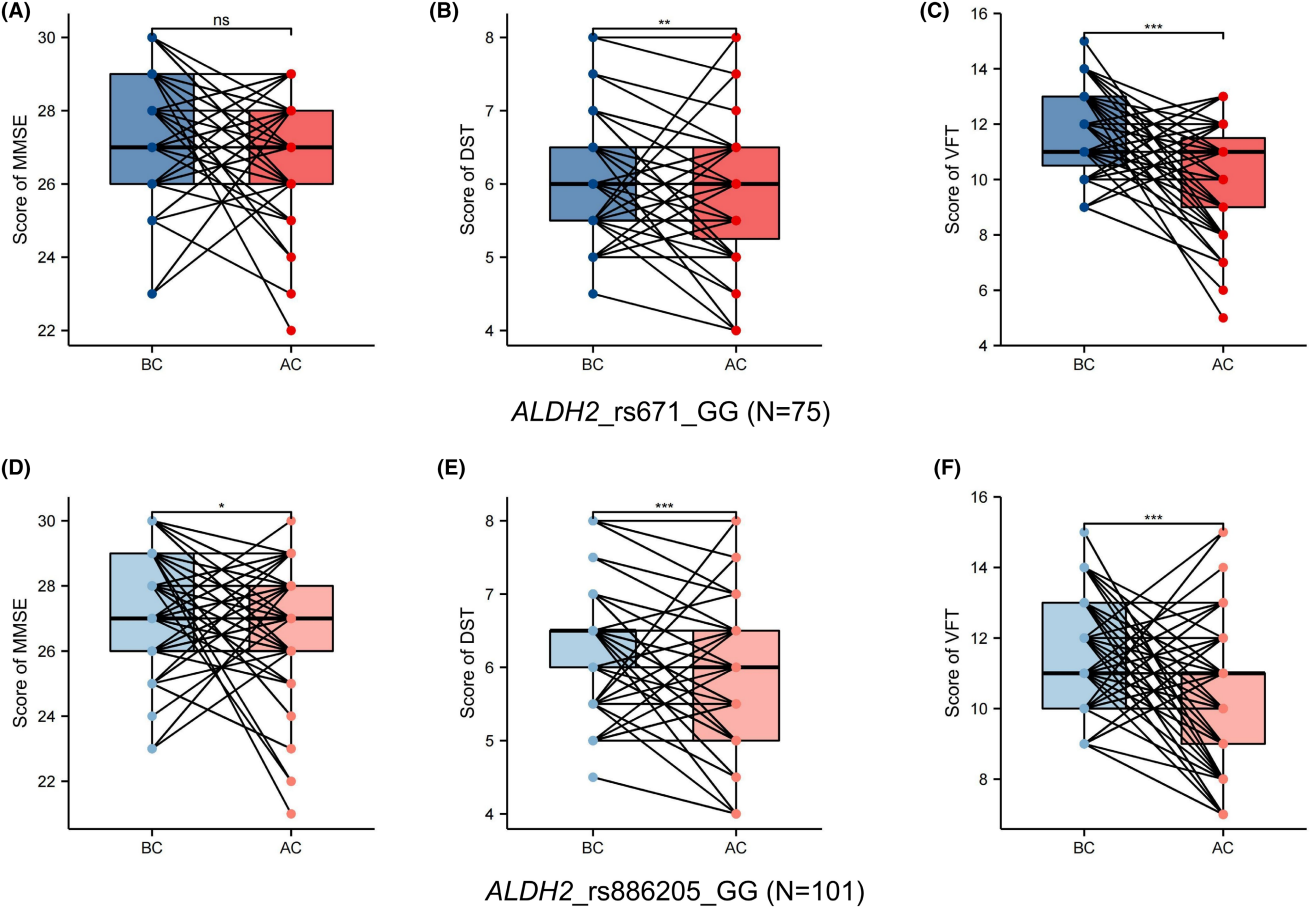
Comparison of cognitive abilities of patients before and after chemotherapy. (A) Score of MMSE of those with *ALDH2*_rs671_GG. (B) Score of DST of those with *ALDH2*_rs671_GG. (C) Score of VFT of those with *ALDH2*_rs671_GG. (D) Score of MMSE of those with *ALDH2*_rs886205_GG. (E) Score of DST of those with *ALDH2*_rs886205_GG. (F) Score of VFT of those with *ALDH2*_rs886205_GG. BC means before chemotherapy, AC means after chemotherapy, *p < 0.05, **p < 0.01, ****p < 0.0001, and *p* < 0.05 were considered significant

The comparison results of cognitive function before and after chemotherapy for different SNPs in *ALDH2*_rs671 are shown in Figure 2 and Table 3. No significant difference was noted in cognitive function between the two groups before chemotherapy. No significant difference was found in cognitive function between the BC and AC groups in rs671(AG/AA) patients. For rs671(GG) patients, the DST and VFT scores after chemotherapy were significantly reduced, suggesting a decline in cognitive ability.

**TABLE 3:** Comparison of cognitive abilities before and after chemotherapy based on rs671 type

rs671	N	MMSE	DST	VFT
Before chemotherapy				
rs671(GG) group	75	27.4 (1.69)	6.1 (0.72)	11.6 (1.46)
rs671(AG/AA) group	49	27.4 (1.51)	6.3 (0.82)	11.3 (1.59)
*t*/z		−0.027	−0.927	0.863
*p*		0.978	0.356	0.390
rs671(GG) patients				
BC group	75	27.4 (1.69)	6.1 (0.72)	11.6 (1.46)
AC group	75	26.9 (1.49)	5.8 (0.85)	10.2 (1.85)
*t*/z		1.919	2.769^a^	4.624
*p*		0.059	0.006**	0.000***
rs671(AG/AA) patients				
BC group	49	27.4 (1.51)	6.3 (0.82)	11.3 (1.59)
AC group	49	26.8 (2.06)	6.0 (0.97)	10.5 (2.20)
*t*/z		1.641	1.979	1.920
*p*		0.107	0.054	0.061

^a^ Wilcoxon signed‐rank test.

** *p* < 0.01.

*** *p* < 0.001.

### 3.3 Cognitive abilities of *ALDH2*_rs886205 patients before and after chemotherapy

The comparison results of cognitive function before and after chemotherapy for different SNPs in *ALDH2*_rs886205 are shown in Figure 2 and Table 4. Before chemotherapy, the difference between the two groups was statistically significant only for the DST score. A statistically significant difference was found in the VFT scores between the BC and AC groups in rs886205 (AG/AA) patients. For rs886205 (GG) patients, the MMSE, DST, and VFT scores after chemotherapy were significantly reduced, suggesting a decline in cognitive ability.

**TABLE 4:** Comparison of cognitive abilities before and after chemotherapy based on rs886205 type

rs886205	N	MMSE	DST	VFT
Before chemotherapy				
rs886205 (GG) group	101	27.4 (1.69)	6.27 (0.78)	11.5 (1.51)
rs886205 (AG/AA) group	23	27.5 (1.27)	5.87 (0.57)	11.6 (1.53)
*t*/z		−0.216	2.346	−0.409
*p*		0.806	0.016^*^	0.683
rs886205 (GG) patients				
BC group	101	27.4 (1.69)	6.27 (0.78)	11.5 (1.51)
AC group	101	26.8 (1.73)	5.92 (0.93)	10.4 (1.91)
*t*/z		2.405	3.663^a^	4.709
*p*		0.018^*^	0.000***	0.000***
rs886205 (AG/AA) patients				
BC group	23	27.5 (1.27)	5.87 (0.57)	11.6 (1.53)
AC group	23	27.0 (1.74)	5.83 (0.73)	10.2 (2.37)
*t*/z		0.828	0.253	2.273
*p*		0.417	0.803	0.033^*^

^a^ Wilcoxon signed‐rank test.

* *p* < 0.05.

*** *p* < 0.001.

### 3.4 Cognitive abilities of *ALDH2*_rs4648328 patients before and after chemotherapy

**FIGURE 3:**
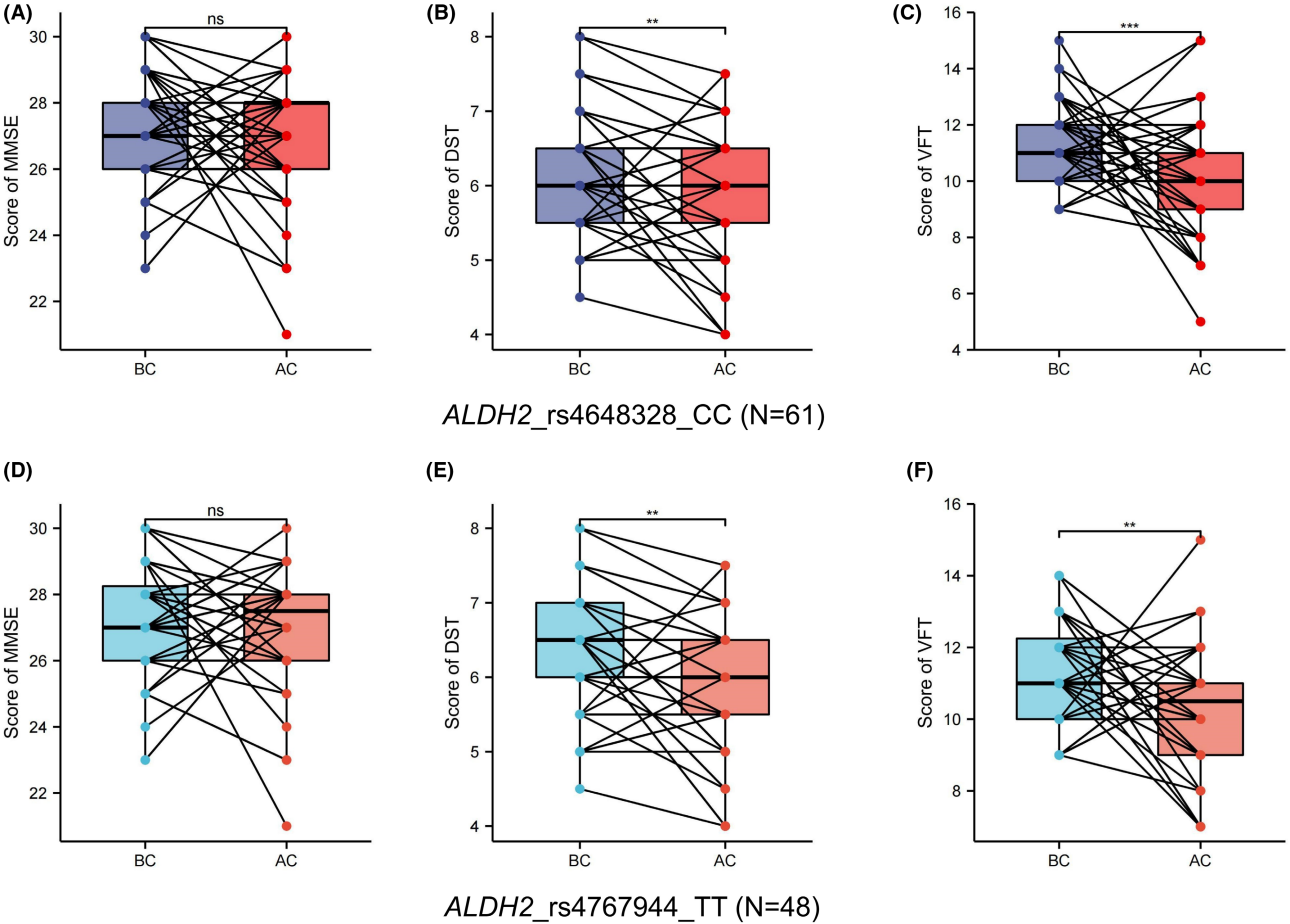
Comparison of cognitive abilities of patients before and after chemotherapy. (A) Score of MMSE of those with *ALDH2*_rs4648328_CC. (B) Score of DST of those with *ALDH2*_rs4648328_CC. (C) Score of VFT of those with *ALDH2*_rs4648328_CC. (D) Score of MMSE of those with *ALDH2*_rs4767944_TT. (E) Score of DST of those with *ALDH2*_rs4767944_TT. (F) Score of VFT of those with *ALDH2*_rs4767944_TT. BC means before chemotherapy, AC means after chemotherapy, *p < 0.05, **p < 0.01, ****p < 0.0001, and *p* < 0.05 were considered significant.

The comparison results of cognitive function before and after chemotherapy for different SNPs in *ALDH2*_rs4648328 are shown in Figure 3 and Table 5. No significant difference was observed in cognitive function between the two groups. A statistically significant difference was noted in the VFT scores between the BC and AC groups in rs4648328 (CT/TT) patients. For rs4648328 (CC) patients, the DST and VFT scores after chemotherapy were significantly reduced, suggesting a decline in cognitive ability.

**TABLE 5:** Comparison of cognitive abilities before and after chemotherapy based on rs4648328 type

rs4648328	N	MMSE	DST	VFT
Before chemotherapy				
rs4648328 (CC) group	61	27.2 (1.68)	6.20 (0.78)	11.4 (1.46)
rs4648328 (CT/TT) group	63	27.6 (1.54)	6.19 (0.74)	11.6 (1.56)
*t*/z		−1.291	0.106	−0.594
*p*		0.199	0.916	0.554
rs4648328 (CC) patients				
BC group	61	27.2 (1.68)	6.20 (0.78)	11.41 (1.46)
AC group	61	27.0 (1.81)	5.84 (0.86)	10.20 (1.91)
*t*/z		0.648	2.850^a^	3.477
*p*		0.520	0.004**	0.001**
rs4648328 (CT/TT) patients				
BC group	63	27.6 (1.54)	6.19 (0.74)	11.6 (1.56)
AC group	63	26.7 (1.64)	5.96 (0.93)	10.5 (2.08)
*t*/z		2.859^a^	1.806	3.136
*p*		0.004**	0.076	0.003**

^a^ Wilcoxon signed‐rank test.

** *p* < 0.01.

### 3.5 Cognitive abilities of *ALDH2*_rs4767944 patients before and after chemotherapy

The comparison results of cognitive function before and after chemotherapy for different SNPs in *ALDH2*_rs4767944 are shown in Figure 3 and Table 6. No significant difference was found in cognitive function between the two groups. A statistically significant difference was observed in the VFT scores between the BC and AC groups in rs4767944 (CT/CC) patients. For rs4767944 (TT) patients, the DST and VFT scores after chemotherapy were significantly reduced, suggesting a decline in cognitive ability.

**TABLE 6:** Comparison of cognitive abilities before and after chemotherapy based on rs4767944 type

rs4767944	N	MMSE	DST	VFT
Before chemotherapy				
rs4767944 (TT) group	48	27.2 (1.81)	6.31 (0.80)	11.4 (1.44)
rs4767944 (CT/CC) group	76	27.5 (1.48)	6.10 (0.72)	11.6 (1.56)
*t*/z		−1.109	1.418	−0.619
*p*		0.270	0.159	0.537
rs4767944 (TT) patients				
BC group	48	27.2 (1.81)	6.31 (0.80)	11.4 (1.44)
AC group	48	27.0 (1.80)	5.89 (0.89)	10.3 (1.74)
*t*/z		0.669	2.967^a^	2.776
*p*		0.507	0.003**	0.008**
rs4767944 (CT/CC) patients				
BC group	76	27.5 (1.48)	6.10 (0.72)	11.6 (1.56)
AC group	76	26.8 (1.68)	5.90 (0.91)	10.3 (2.15)
*t*/z		2.560^a^	1.878	3.757
*p*		0.010	0.064	0.000***

^a^ Wilcoxon signed‐rank test.

** *p* < 0.01.

*** *p* < 0.001.

### 3.6 Correlation analysis of cognitive outcomes in patients with SNPs characteristic of cognitive decline after chemotherapy

**FIGURE 4:**
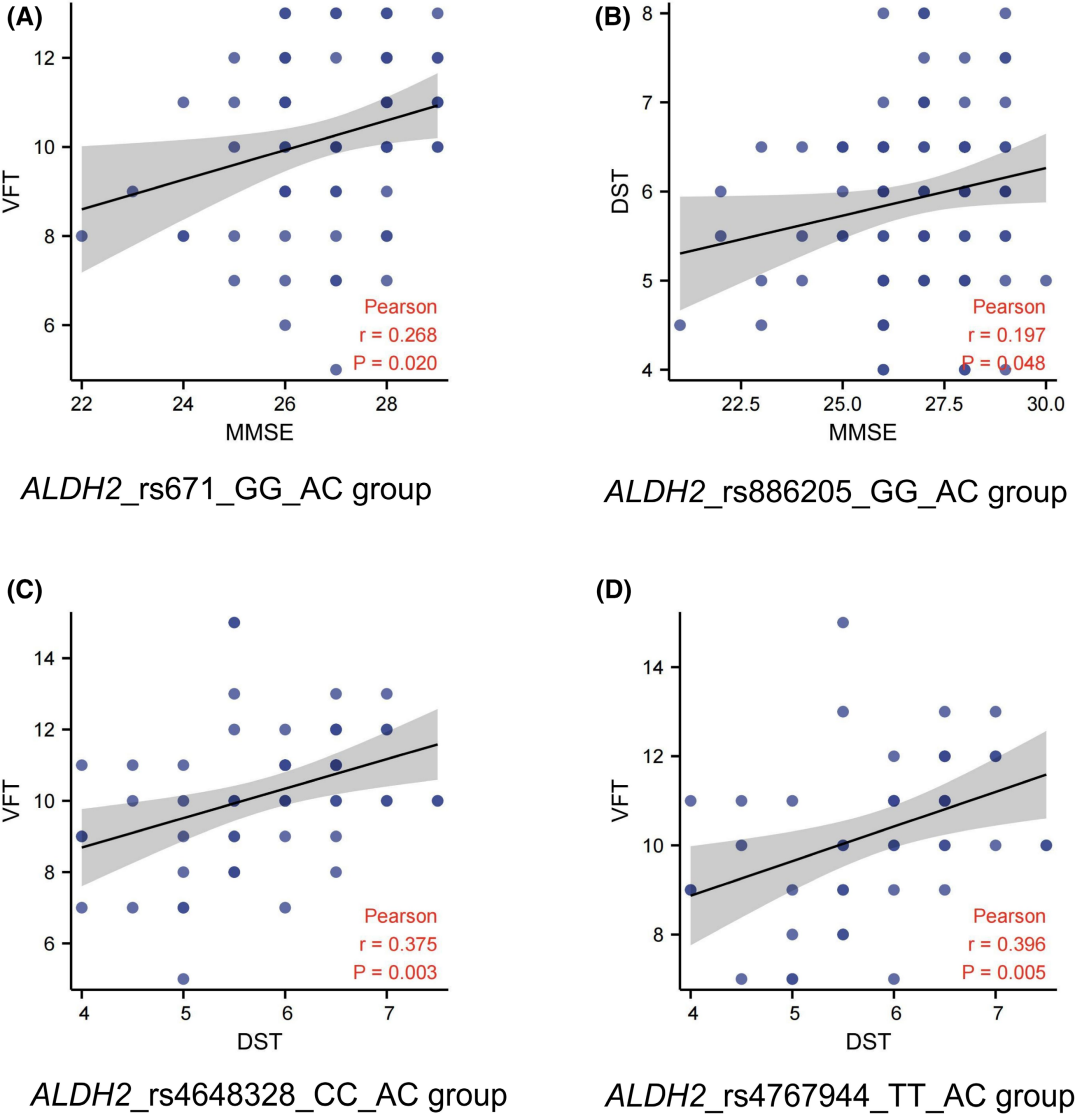
Correlation analysis of cognitive outcomes in patients with single‐nucleotide polymorphisms characteristic of cognitive decline after chemotherapy. (A) Correlation between MMSE and VFT in *ALDH2*_rs671_GG patients after chemotherapy. (B) Correlation between MMSE and DST in *ALDH2*_rs886205_GG patients after chemotherapy. (C) Correlation between DST and VFT in *ALDH2*_rs4648328_CC patients after chemotherapy. (D) Correlation between DST and VFT in *ALDH2*_rs4767944_TT patients after chemotherapy

A significant positive correlation exists between MMSE and VFT scores in patients with *ALDH2*_rs671_GG after chemotherapy (Figure 4A). A significant positive correlation was found between the MMSE and DST scores in patients with *ALDH2*_rs886205_GG after chemotherapy (Figure 4B). A significant positive correlation was observed between DST and VFT in patients with *ALDH2*_rs4648328_CC after chemotherapy (Figure 4C). A significant positive correlation was observed between DST and VFT in patients with *ALDH2*_rs4767944_TT after chemotherapy (Figure 4D).

### 3.7 The expression of ALDH2 in the brain

**FIGURE 5:**
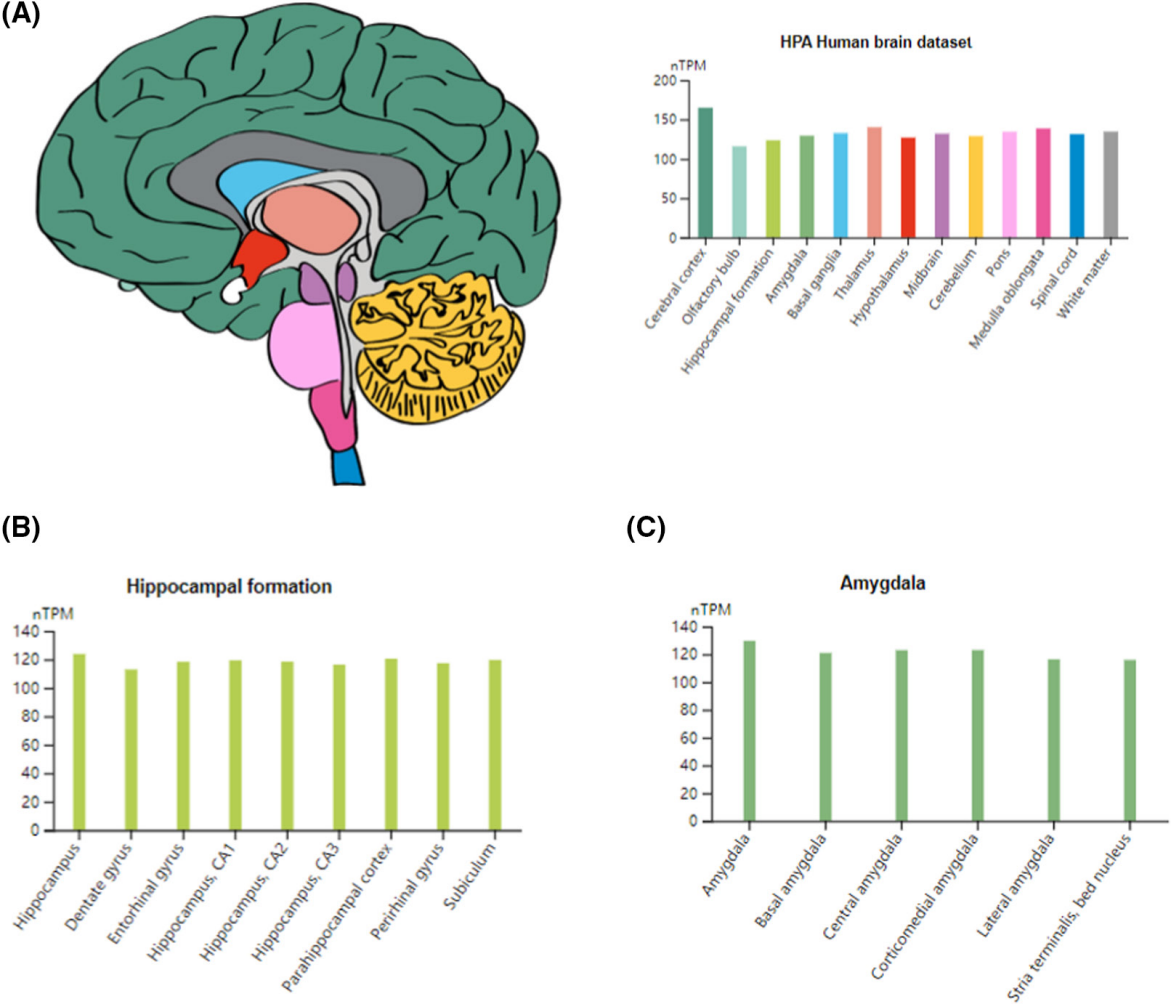
Expression of *ALDH2* in the brain. (A) Expression of *ALDH2* in different parts of the brain. (B) Expression of *ALDH2* in different parts of the hippocampal formation. (C) Expression of *ALDH2* in different parts of the amygdala

*ALDH2* expression differs in different parts of the brain. The highest expression was observed in the cerebral cortex and the lowest in the olfactory bulb. We further analysed the expression of *ALDH2* in hippocampal formation and amygdala, and the results are shown in Figure 5.

## 4 DISCUSSION

This study explored the decline in cognitive function in patients with different molecular types of *ALDH2* after chemotherapy and found that patients with rs671_GG, rs886205_GG, rs4648328_CC, and rs4767944_TT were more likely to have cognitive decline after chemotherapy. This method can help identify the population susceptible to cognitive decline in chemotherapy and help these patients by providing early intervention to improve their QoL, which is meaningful.

As we know, the *ALDH2* gene is a key gene for aldehyde metabolism.^42^ In addition, aldehydes can damage the nerve cells in the brain.^43^, ^44^, ^45^ Therefore, one of the possible reasons for cognitive impairment in patients undergoing chemotherapy is the accumulation of aldehydes in the body and nervous system. There are common polymorphisms of *ALDH2* in Asians: *ALDH2*_rs671 (AG/AA) and wild‐type *ALDH2*_rs671 (GG). These two genotypes are almost halved in the Asian population.^19^ Studies have found that patients with an inactivating point gene polymorphism coding for *ALDH2*_rs671 (AG/AA) are markedly protected against alcoholism. Cognitive impairment caused due to alcohol is similar to that caused by chemotherapy.^46^, ^47^ Interestingly, our results indicated that patients with *ALDH2*_rs671 (AG/AA) had no significant changes in cognitive function after chemotherapy, while those with *ALDH2*_rs671 (GG) showed a significant cognitive decline (Table 2). Therefore, we propose that patients with *ALDH2*_rs671 (GG) may be more susceptible to CRCI. Such patients should receive early cognitive interventions to help improve their QoL.

To improve the sensitivity of CRCI‐susceptible population discrimination, we analysed three other common *ALDH2* alleles: rs886205, rs4648328, and rs4767944. These polymorphism sites also correlate with cognitive function and can be used to identify Alzheimer's disease,^48^, ^49^ alcohol‐related cognitive impairment, and other diseases.^50^, ^51^ We found that patients with breast cancer with *ALDH2*_rs886205_GG, *ALDH2*_rs4648328_CC, and *ALDH2*_rs4767944_TT also showed a significant cognitive decline after chemotherapy (TABLE 3‐5). A possible reason is that patients with different *ALDH2* genotypes have different abilities to metabolise aldehydes.^52^ Therefore, there are differences in the degree of cognitive impairment caused by aldehyde accumulation during chemotherapy.^53^, ^54^

Recent studies have confirmed that *ALDH2* in astrocytes is involved in the regulation of behavioural and cognitive functions related to ethanol metabolism in the brain.^55^ Both chemotherapy‐ and ethanol metabolism‐induced cognitive impairment can be caused by aldehydes^42^, ^56^; thus, *ALDH2* in the brain may also affect CRCI by participating in aldehyde regulation. In this study, we analysed the expression of *ALDH2* in the brain, hippocampal formation, and amygdala based on the HPA database. In future studies, it may be possible to improve and prevent CRCI by regulating the function of *ALDH2* in the brain, which will greatly enhance the QoL of patients with cancer undergoing chemotherapy.

The limitations of this study include the following aspects that need to be addressed in future studies. Patients with both primary and metastatic breast cancer were enrolled in this study. This heterogeneity of the sample will affect the persuasiveness of the results. This was a cross‐sectional study, and future studies should include a control group and conduct long‐term follow‐ups. Our study focused on Asian populations. Although Asians have a higher mutation rate of *ALDH2*, aldehyde detoxification of *ALDH2* is also worthy of investigation in other ethnic groups with CRCI. The MMSE lacks sensitivity for CRCI, and more effective cognitive testing methods should be used in future research. Repeated measurements will increase the results' accuracy. It would be meaningful to measure cognitive function at different time points after chemotherapy to explore the correlation between CRCI symptoms and the time after chemotherapy.

## 5 CONCLUSION

**FIGURE 6:**
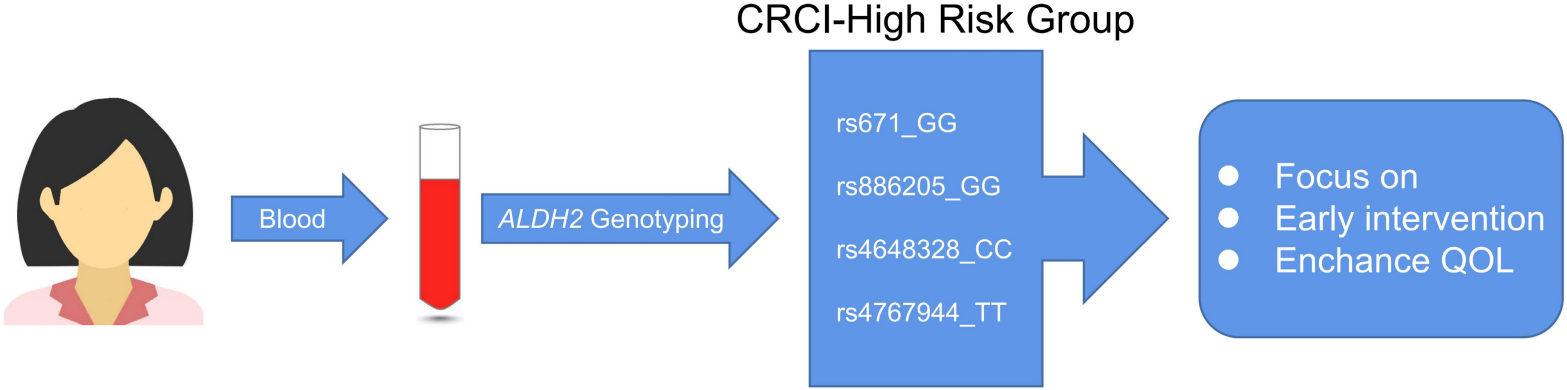
Scientific value of this research

Since breast cancer CRCI is very common and markedly affects patients' QoL, it is clinically meaningful to identify which patients are more likely to develop CRCI.^37^, ^57^ This study indicated that patients with *ALDH2* rs671_GG, rs886205_GG, rs4648328_CC, and rs4767944_TT polymorphisms were more likely to suffer from cognitive impairment during chemotherapy. For these patients, their cognitive status should be monitored while paying attention to somatic side effects to improve QoL (Figure 6). More importantly, as *ALDH2* rs671 is a common polymorphism site in Asians, this study has important implications for Asian women with breast cancer. Broadly, this study highlighted the direction for the study of *ALDH2* in CRCI; however, the specific mechanism can be explored through animal experiments in the future.

## AUTHOR CONTRIBUTIONS

**Senbang Yao:** Conceptualization (lead); formal analysis (lead); investigation (lead); resources (lead); software (lead); writing – original draft (lead). **Wen Li:** Conceptualization (equal); data curation (equal); investigation (equal); resources (equal). **Shaochun Liu:** Formal analysis (equal); investigation (equal); supervision (equal); writing – original draft (equal). **Yinlian Cai:** Data curation (supporting); supervision (supporting). **Qianqian Zhang:** Formal analysis (supporting); methodology (supporting); resources (supporting). **Lingxue Tang:** Data curation (supporting); investigation (supporting); visualization (supporting). **Sheng Yu:** Data curation (supporting); supervision (supporting); writing – review and editing (supporting). **Yanyan Jing:** Data curation (supporting); methodology (supporting); project administration (supporting). **Xiangxiang Yin:** Data curation (supporting); funding acquisition (supporting); validation (supporting). **Huaidong Cheng:** Conceptualization (lead); funding acquisition (lead); writing – review and editing (lead).

## FUNDING INFORMATION

This study was supported by The National Natural Science Foundation of China (Nos. 81872504 and 81372487).

## CONFLICT OF INTEREST

The authors declare there are no competing interests.

## ETHICS APPROVAL

All procedures involving human participants were in accordance with the ethical standards of the institutional and/or national research committee and with the 1964 Helsinki Declaration and its later amendments or comparable ethical standards. The study was approved by the Research Ethics Committee of the Second Affiliated Hospital of Anhui Medical University (approval number:2012088), and all patients provided written informed consent.

## Supporting information


Figures S1‐S2

Data S1

Data S2

## Data Availability

The data of this study will be available from the correspondent on reasonable request.

## References

[1] Siegel RL , Miller KD , Fuchs HE , Jemal A . CA Cancer J Clin. 2022. 2022;72(1):7–33. doi:10.3322/caac.21708 35020204

[2] Chen J , Haanpää MK , Gruber JJ , Jäger N , Ford JM , Snyder MP . High‐resolution bisulfite‐sequencing of peripheral blood DNA methylation in early‐onset and familial risk breast cancer patients. Clinical Cancer Research: An Official Journal of the American Association for Cancer Research. 2019;25(17):5301–14. Epub 2019/06/09. PubMed PMID: 31175093; PubMed Central PMCID: PMCPMC6726519. doi:10.1158/1078-0432.Ccr-18-2423 31175093 PMC6726519

[3] Bray F , Ferlay J , Soerjomataram I , Siegel RL , Torre LA , Jemal A . Global cancer statistics 2018: GLOBOCAN estimates of incidence and mortality worldwide for 36 cancers in 185 countries. CA Cancer J Clin 2018;68(6):394–424. Epub 2018/09/13. PubMed PMID: 30207593. doi:10.3322/caac.21492 30207593

[4] Chen W , Zheng R , Baade PD , Zhang S , Zeng H , Bray F , et al. Cancer statistics in China, 2015. CA Cancer J Clin 2016;66(2):115–32. Epub 2016/01/26. PubMed PMID: 26808342. doi:10.3322/caac.21338 26808342

[5] Cao W , Chen HD , Yu YW , Li N , Chen WQ . Changing profiles of cancer burden worldwide and in China: a secondary analysis of the global cancer statistics 2020. Chin Med J (Engl). 2021;134(7):783–91. Epub 2021/03/19. PubMed PMID: 33734139; PubMed Central PMCID: PMCPMC8104205. doi:10.1097/cm9.0000000000001474 33734139 PMC8104205

[6] An J , Zhou K , Li M , Li X . Assessing the relationship between body image and quality of life among rural and urban breast cancer survivors in China. BMC women's Health. 2022;22(1):61. Epub 2022/03/06. PubMed PMID: 35246115; PubMed Central PMCID: PMCPMC8896367. doi:10.1186/s12905-022-01635-y 35246115 PMC8896367

[7] Provenzano E. Neoadjuvant chemotherapy for breast cancer: moving beyond pathological complete response in the molecular age. Acta Medica Academica 2021;50(1):88–109. Epub 2021/06/03. PubMed PMID: 34075766. doi:10.5644/ama2006-124.328 34075766

[8] Dieci MV , Griguolo G , Bottosso M , Tsvetkova V , Giorgi CA , Vernaci G , et al. Impact of estrogen receptor levels on outcome in non‐metastatic triple negative breast cancer patients treated with neoadjuvant/adjuvant chemotherapy. NPJ Breast Cancer. 2021;7(1):101. Epub 2021/08/04. PubMed PMID: 34341356. doi:10.1038/s41523-021-00308-7 34341356 PMC8329161

[9] Hauner K , Maisch P , Retz M . [Side effects of chemotherapy]. Der Urologe Ausg A 2017;56(4):472–9. Epub 2017/03/03. PubMed PMID: 28251254. doi:10.1007/s00120-017-0338-z 28251254

[10] Lv L , Mao S , Dong H , Hu P , Dong R . Pathogenesis, assessments, and Management of Chemotherapy‐Related Cognitive Impairment (CRCI): An updated literature review. J Oncol. 2020;2020:3942439.1–11. Epub 2020/07/21. PubMed PMID: 32684930; PubMed Central PMCID: PMCPMC7333028. doi:10.1155/2020/3942439 PMC7333028 32684930

[11] Oberste M , Schaffrath N , Schmidt K , Bloch W , Jäger E , Steindorf K , et al. Protocol for the "Chemobrain in motion ‐ study" (CIM ‐ study): a randomized placebo‐controlled trial of the impact of a high‐intensity interval endurance training on cancer related cognitive impairments in women with breast cancer receiving first‐line chemotherapy. BMC Cancer. 2018;18(1):1071. Epub 2018/11/08. PubMed PMID: 30400840; PubMed Central PMCID: PMCPMC6220507. doi:10.1186/s12885-018-4992-3 30400840 PMC6220507

[12] Sousa H , Almeida S , Bessa J , Pereira MG . The developmental trajectory of cancer‐related cognitive impairment in breast cancer patients: a systematic review of longitudinal neuroimaging studies. Neuropsychol Rev 2020;30(3):287–309. Epub 2020/07/02. PubMed PMID: 32607817. doi:10.1007/s11065-020-09441-9 32607817

[13] Protas PT , Muszynska‐Roslan K , Holownia A , Grabowska A , Wielgat P , Krawczuk‐Rybak M , et al. Negative correlation between cerebrospinal fluid tau protein and cognitive functioning in children with acute lymphoblastic leukemia. Pediatr Blood Cancer 2009;53(1):105–8. Epub 2009/03/25. PubMed PMID: 19309718. doi:10.1002/pbc.22029 19309718

[14] Fernandez HR , Varma A , Flowers SA , Rebeck GW . Cancer chemotherapy related cognitive impairment and the impact of the Alzheimer's disease risk factor APOE. Cancers (Basel). 2020;12(12):3842. Epub 2020/12/24. PubMed PMID: 33352780; PubMed Central PMCID: PMCPMC7766535. doi:10.3390/cancers12123842 33352780 PMC7766535

[15] Battaglia V , DeStefano Shields C , Murray‐Stewart T , Casero RA, Jr. Polyamine catabolism in carcinogenesis: potential targets for chemotherapy and chemoprevention. Amino Acids. 2014;46(3):511–9. Epub 2013/06/19. PubMed PMID: 23771789; PubMed Central PMCID: PMCPMC3795954. doi:10.1007/s00726-013-1529-6 23771789 PMC3795954

[16] Li M , Zhang P , Wei HJ , Li MH , Zou W , Li X , et al. Hydrogen sulfide ameliorates homocysteine‐induced cognitive dysfunction by inhibition of reactive aldehydes involving upregulation of ALDH2. Int J Neuropsychopharmacol. 2017;20(4):305–15. Epub 2016/12/19. PubMed PMID: 27988490; PubMed Central PMCID: PMCPMC5409037. doi:10.1093/ijnp/pyw103 27988490 PMC5409037

[17] Juan Z , Chen J , Ding B , Yongping L , Liu K , Wang L , et al. Probiotic supplement attenuates chemotherapy‐related cognitive impairment in patients with breast cancer: a randomised, double‐blind, and placebo‐controlled trial. Eur J Cancer (Oxford, England: 1990). 2022;161:10–22. Epub 2021/12/14. PubMed PMID: 34896904. doi:10.1016/j.ejca.2021.11.006 34896904

[18] Cook WK , Tam CC , Luczak SE , Kerr WC , Mulia N , Lui C , et al. Alcohol consumption, cardiovascular‐related conditions, and ALDH2*2 ethnic group prevalence in Asian Americans. Alcohol, Clin Exp Res. 2021;45(2):418–28. Epub 2020/12/23. PubMed PMID: 33349921; PubMed Central PMCID: PMCPMC8545194. doi:10.1111/acer.14539 33349921 PMC8545194

[19] Liangpunsakul S , Haber P , McCaughan GW . Alcoholic liver disease in Asia, Europe, and North America. Gastroenterology. 2016;150(8):1786–97. Epub 2016/03/01. PubMed PMID: 26924091; PubMed Central PMCID: PMCPMC4887319. doi:10.1053/j.gastro.2016.02.043 26924091 PMC4887319

[20] Hung CL , Sung KT , Chang SC , Liu YY , Kuo JY , Huang WH , et al. Variant aldehyde dehydrogenase 2 (ALDH2*2) as a risk factor for mechanical LA substrate formation and atrial fibrillation with modest alcohol consumption in ethnic Asians. Biomolecules. 2021;11(11):1559. Epub 2021/11/28. PubMed PMID: 34827557; PubMed Central PMCID: PMCPMC8615757. doi:10.3390/biom11111559 34827557 PMC8615757

[21] Wang LS , Wu ZX . ALDH2 and cancer therapy. Adv Exp Med Biol 2019;1193:221–8. Epub 2019/08/02. PubMed PMID: 31368107. doi:10.1007/978-981-13-6260-6_13 31368107

[22] Chen CH , Ferreira JCB , Mochly‐Rosen D . ALDH2 and cardiovascular disease. Adv Exp Med Biol 2019;1193:53–67. Epub 2019/08/02. PubMed PMID: 31368097. doi:10.1007/978-981-13-6260-6_3 31368097

[23] Yang Y , Chen W , Wang X , Ge W . Impact of mitochondrial aldehyde dehydrogenase 2 on cognitive impairment in the AD model mouse. Acta Biochim Biophys Sin 2021;53(7):837–47. Epub 2021/05/07. PubMed PMID: 33954430. doi:10.1093/abbs/gmab057 33954430

[24] Yu RL , Tan CH , Lu YC , Wu RM . Aldehyde dehydrogenase 2 is associated with cognitive functions in patients with Parkinson's disease. Sci Rep. 2016;6:30424. Epub 2016/07/28. PubMed PMID: 27453488; PubMed Central PMCID: PMCPMC4958972. doi:10.1038/srep30424 27453488 PMC4958972

[25] Tan T , Zhang Y , Luo W , Lv J , Han C , Hamlin JNR , et al. Formaldehyde induces diabetes‐associated cognitive impairments. FASEB J 2018;32(7):3669–79. Epub 2018/02/07. PubMed PMID: 29401634. doi:10.1096/fj.201701239R 29401634

[26] Kim J , Chen CH , Yang J , Mochly‐Rosen D . Aldehyde dehydrogenase 2*2 knock‐in mice show increased reactive oxygen species production in response to cisplatin treatment. J Biomed Sci. 2017;24(1):33. Epub 2017/05/24. PubMed PMID: 28532411; PubMed Central PMCID: PMCPMC5439151. doi:10.1186/s12929-017-0338-8 28532411 PMC5439151

[27] Rostami A , Taleahmad F , Haddadzadeh‐Niri N , Joneidi E , Afshin‐Majd S , Baluchnejadmojarad T , et al. Sinomenine attenuates Trimethyltin‐induced cognitive decline via targeting hippocampal oxidative stress and neuroinflammation. J Mol Neurosci 2022; 72(8):1609–1621. Epub 2022/05/12. PubMed PMID: 35543800. doi:10.1007/s12031-022-02021-x 35543800

[28] Cheng H , Li W , Gan C , Zhang B , Jia Q , Wang K . The COMT (rs165599) gene polymorphism contributes to chemotherapy‐induced cognitive impairment in breast cancer patients. Am J Transl Res. 2016;8(11):5087–97. Epub 2016/12/03. PubMed PMID: 27904710; PubMed Central PMCID: PMCPMC5126352. 27904710 PMC5126352

[29] Park JY , Lengacher CA , Reich RR , Park HY , Whiting J , Nguyen AT , et al. Translational genomic research: the association between genetic profiles and cognitive functioning or cardiac function among breast cancer survivors completing chemotherapy. Biol Res Nurs 2022; 24(4):433–447.10998004221094386. Epub 2022/05/03. PubMed PMID: 35499926. doi:10.1177/10998004221094386 35499926 PMC9630728

[30] Tan PH , Ellis I , Allison K , Brogi E , Fox SB , Lakhani S , et al. The 2019 World Health Organization classification of tumours of the breast. Histopathology 2020;77(2):181–5. Epub 2020/02/15. PubMed PMID: 32056259. doi:10.1111/his.14091 32056259

[31] Giuliano AE , Connolly JL , Edge SB , Mittendorf EA , Rugo HS , Solin LJ , et al. Breast cancer‐major changes in the American joint committee on cancer eighth edition cancer staging manual. CA Cancer J Clin 2017;67(4):290–303. Epub 2017/03/16. PubMed PMID: 28294295. doi:10.3322/caac.21393 28294295

[32] Gradishar WJ , Moran MS , Abraham J , Aft R , Agnese D , Allison KH , et al. NCCN guidelines® insights: breast cancer, version 4.2021. J Natl Compr Canc Netw 2021;19(5):484–93. Epub 2021/11/19. PubMed PMID: 34794122. doi:10.6004/jnccn.2021.0023 34794122

[33] Burstein HJ , Curigliano G , Thürlimann B , Weber WP , Poortmans P , Regan MM , et al. Customizing local and systemic therapies for women with early breast cancer: the St. Gallen international consensus guidelines for treatment of early breast cancer 2021. Ann Oncol 2021;32(10):1216–35. Epub 2021/07/10. PubMed PMID: 34242744. doi:10.1016/j.annonc.2021.06.023 34242744 PMC9906308

[34] Uhlen M , Zhang C , Lee S , Sjöstedt E , Fagerberg L , Bidkhori G , et al. A pathology atlas of the human cancer transcriptome. Science 2017;357(6352):eaan2507. PubMed PMID: 28818916. doi:10.1126/science.aan2507 28818916

[35] Khan MA , Garg K , Bhurani D , Agarwal NB . Early manifestation of mild cognitive impairment in B‐cell non‐Hodgkin's lymphoma patients receiving CHOP and rituximab‐CHOP chemotherapy. Naunyn Schmiedebergs Arch Pharmacol 2016;389(12):1253–65. Epub 2016/08/29. PubMed PMID: 27568029. doi:10.1007/s00210-016-1290-y 27568029

[36] Dos Santos M , Licaj I , Bellera C , Cany L , Binarelli G , Soubeyran P , et al. Cognitive impairment in older cancer patients treated with first‐line chemotherapy. Cancers (Basel). 2021;13(24):6171. Epub 2021/12/25. PubMed PMID: 34944791; PubMed Central PMCID: PMCPMC8699230. doi:10.3390/cancers13246171 34944791 PMC8699230

[37] Oh PJ , Kim JH . [Chemotherapy‐related cognitive impairment and quality of life in people with colon cancer: the mediating effect of psychological distress]. J Korean Acad Nurs 2016;46(1):19–28. Epub 2016/03/11. PubMed PMID: 26963411. doi:10.4040/jkan.2016.46.1.19 26963411

[38] Hira A , Yabe H , Yoshida K , Okuno Y , Shiraishi Y , Chiba K , et al. Variant ALDH2 is associated with accelerated progression of bone marrow failure in Japanese Fanconi anemia patients. Blood. 2013;122(18):3206–9. Epub 2013/09/17. PubMed PMID: 24037726; PubMed Central PMCID: PMCPMC3953058. doi:10.1182/blood-2013-06-507962 24037726 PMC3953058

[39] Lin CY , Yu RL , Wu RM , Tan CH . Effect of ALDH2 on sleep disturbances in patients with Parkinson's disease. Sci Rep. 2019;9(1):18950. Epub 2019/12/14. PubMed PMID: 31831791; PubMed Central PMCID: PMCPMC6908732. doi:10.1038/s41598-019-55427-w 31831791 PMC6908732

[40] Takeno K , Tamura Y , Kakehi S , Kaga H , Kawamori R , Watada H . ALDH2 rs671 is associated with elevated FPG, reduced glucose clearance and hepatic insulin resistance in Japanese men. J Clin Endocrinol Metab 2021;106(9):e3573‐e81. Epub 2021/05/12. PubMed PMID: 33974068. doi:10.1210/clinem/dgab324 33974068

[41] Yu RL , Tu SC , Wu RM , Lu PA , Tan CH . Interactions of COMT and ALDH2 genetic polymorphisms on symptoms of Parkinson's disease. Brain Sci. 2021;11(3):361. Epub 2021/04/04. PubMed PMID: 33808974; PubMed Central PMCID: PMCPMC8001371. doi:10.3390/brainsci11030361 33808974 PMC8001371

[42] Seo W , Gao Y , He Y , Sun J , Xu H , Feng D , et al. ALDH2 deficiency promotes alcohol‐associated liver cancer by activating oncogenic pathways via oxidized DNA‐enriched extracellular vesicles. J Hepatol. 2019;71(5):1000–11. Epub 2019/07/08. PubMed PMID: 31279903; PubMed Central PMCID: PMCPMC6801025. doi:10.1016/j.jhep.2019.06.018 31279903 PMC6801025

[43] Herr SA , Shi L , Gianaris T , Jiao Y , Sun S , Race N , et al. Critical role of mitochondrial aldehyde dehydrogenase 2 in acrolein sequestering in rat spinal cord injury. Neural Regeneration Research. 2022;17(7):1505–11. Epub 2021/12/18. PubMed PMID: 34916435; PubMed Central PMCID: PMCPMC8771087. doi:10.4103/1673-5374.330613 34916435 PMC8771087

[44] Caruso G , Godos J , Privitera A , Lanza G , Castellano S , Chillemi A , et al. Phenolic acids and prevention of cognitive decline: polyphenols with a neuroprotective role in cognitive disorders and Alzheimer's disease. Nutrients 2022;14(4):819. Epub 2022/02/27. PubMed PMID: 35215469. doi:10.3390/nu14040819 35215469 PMC8875888

[45] Chen C , Lu J , Peng W , Mak MS , Yang Y , Zhu Z , et al. Acrolein, an endogenous aldehyde induces Alzheimer's disease‐like pathologies in mice: a new sporadic AD animal model. Pharmacol Res 2022;175:106003. Epub 2021/11/29. PubMed PMID: 34838693. doi:10.1016/j.phrs.2021.106003 34838693

[46] Rivera‐Meza M , Quintanilla ME , Tampier L , Mura CV , Sapag A , Israel Y . Mechanism of protection against alcoholism by an alcohol dehydrogenase polymorphism: development of an animal model. FASEB J. 2010;24(1):266–74. Epub 2009/08/28. PubMed PMID: 19710201; PubMed Central PMCID: PMCPMC2797030. doi:10.1096/fj.09-132563 19710201 PMC2797030

[47] Moya M , López‐Valencia L , García‐Bueno B , Orio L . Disinhibition‐like behavior correlates with frontal cortex damage in an animal model of chronic alcohol consumption and thiamine deficiency. Biomedicine 2022;10(2):260. Epub 2022/02/26. PubMed PMID: 35203470. doi:10.3390/biomedicines10020260 PMC8869694 35203470

[48] Wu YY , Lee YS , Liu YL , Hsu WC , Ho WM , Huang YH , et al. Association study of alcohol dehydrogenase and aldehyde dehydrogenase polymorphism with Alzheimer disease in the Taiwanese population. Front Neurosci. 2021;15:625885. Epub 2021/02/09. PubMed PMID: 33551739; PubMed Central PMCID: PMCPMC7862325. doi:10.3389/fnins.2021.625885 33551739 PMC7862325

[49] Dong Q , Ren G , Zhang K , Liu D , Dou Q , Hao D . Genetic polymorphisms of ALDH2 are associated with lumbar disc herniation in a Chinese Han population. Sci Rep. 2018;8(1):13079. Epub 2018/09/01. PubMed PMID: 30166580; PubMed Central PMCID: PMCPMC6117275. doi:10.1038/s41598-018-31491-6 30166580 PMC6117275

[50] Haschemi Nassab M , Rhein M , Hagemeier L , Kaeser M , Muschler M , Glahn A , et al. Impaired regulation of ALDH2 protein expression revealing a yet unknown epigenetic impact of rs886205 on specific methylation of a negative regulatory promoter region in alcohol‐dependent patients. Eur Addict Res 2016;22(2):59–69. Epub 2015/09/05. PubMed PMID: 26339786. doi:10.1159/000381018 26339786

[51] You L , Li C , Zhao J , Wang DW , Cui W . Associations of common variants at ALDH2 gene and the risk of stroke in patients with coronary artery diseases undergoing percutaneous coronary intervention. Medicine (Baltimore). 2018;97(19):e0711. Epub 2018/05/10. PubMed PMID: 29742731; PubMed Central PMCID: PMCPMC5959384. doi:10.1097/md.0000000000010711 29742731 PMC5959384

[52] Crabb DW , Edenberg HJ , Bosron WF , Li TK . Genotypes for aldehyde dehydrogenase deficiency and alcohol sensitivity. The inactive ALDH2(2) allele is dominant. J Clin Invest. 1989;83(1):314–6. Epub 1989/01/01. PubMed PMID: 2562960; PubMed Central PMCID: PMCPMC303676. doi:10.1172/jci113875 2562960 PMC303676

[53] Noël X , Saeremans M , Kornreich C , Chatard A , Jaafari N , D'Argembeau A . Reduced calibration between subjective and objective measures of episodic future thinking in alcohol use disorder. Alcohol Clin Exp Res 2022;46(2):300–11. Epub 2022/02/20. PubMed PMID: 35181906. doi:10.1111/acer.14763 35181906

[54] Bian H , Wu Y , Cui Z , Zheng H , Li Y , Zou D . Study on the autophagy‐related mechanism of puerarin in improving the cognitive impairment induced by alcohol in female mice. Brain Inj 2022; 36(1):137–145. Epub 2022/02/10. PubMed PMID: 35138214. doi:10.1080/02699052.2022.2037712 35138214

[55] Jin S , Cao Q , Yang F , Zhu H , Xu S , Chen Q , et al. Brain ethanol metabolism by astrocytic ALDH2 drives the behavioural effects of ethanol intoxication. Nat Metab. 2021;3(3):337–51. Epub 2021/03/25. PubMed PMID: 33758417; PubMed Central PMCID: PMCPMC8294184. doi:10.1038/s42255-021-00357-z 33758417 PMC8294184

[56] Xue L , Yu D , Wang L , Sun J , Song Y , Jia Y , et al. Selective antitumor activity and photocytotoxicity of glutathione‐activated Abasic site trapping agents. ACS Chem Biol 2022;17(4):797–803. Epub 2022/03/18. PubMed PMID: 35297620. doi:10.1021/acschembio.2c00061 35297620

[57] Syed Alwi SM , Narayanan V , Mohd Taib NA , Che Din N . Chemotherapy‐related cognitive impairment (CRCI) among early‐stage breast cancer survivors in Malaysia. J Clin Exp Neuropsychol 2021;43(5):534–45. Epub 2021/08/10. PubMed PMID: 34369307. doi:10.1080/13803395.2021.1945539 34369307

