# Supplementary material for: Aldehyde dehydrogenase 2 polymorphism is associated with chemotherapy‐related cognitive impairment in patients with breast cancer who receive chemotherapy
Source: Cancer Med. 2022 Oct 6;12(5):5209–21. doi: 10.1002/cam4.5319 (PMC10028021; doi:10.1002/cam4.5319)
Supplement: Supplementary file 1 — Figures S1‐S2 [file CAM4-12-5209-s002.pdf]

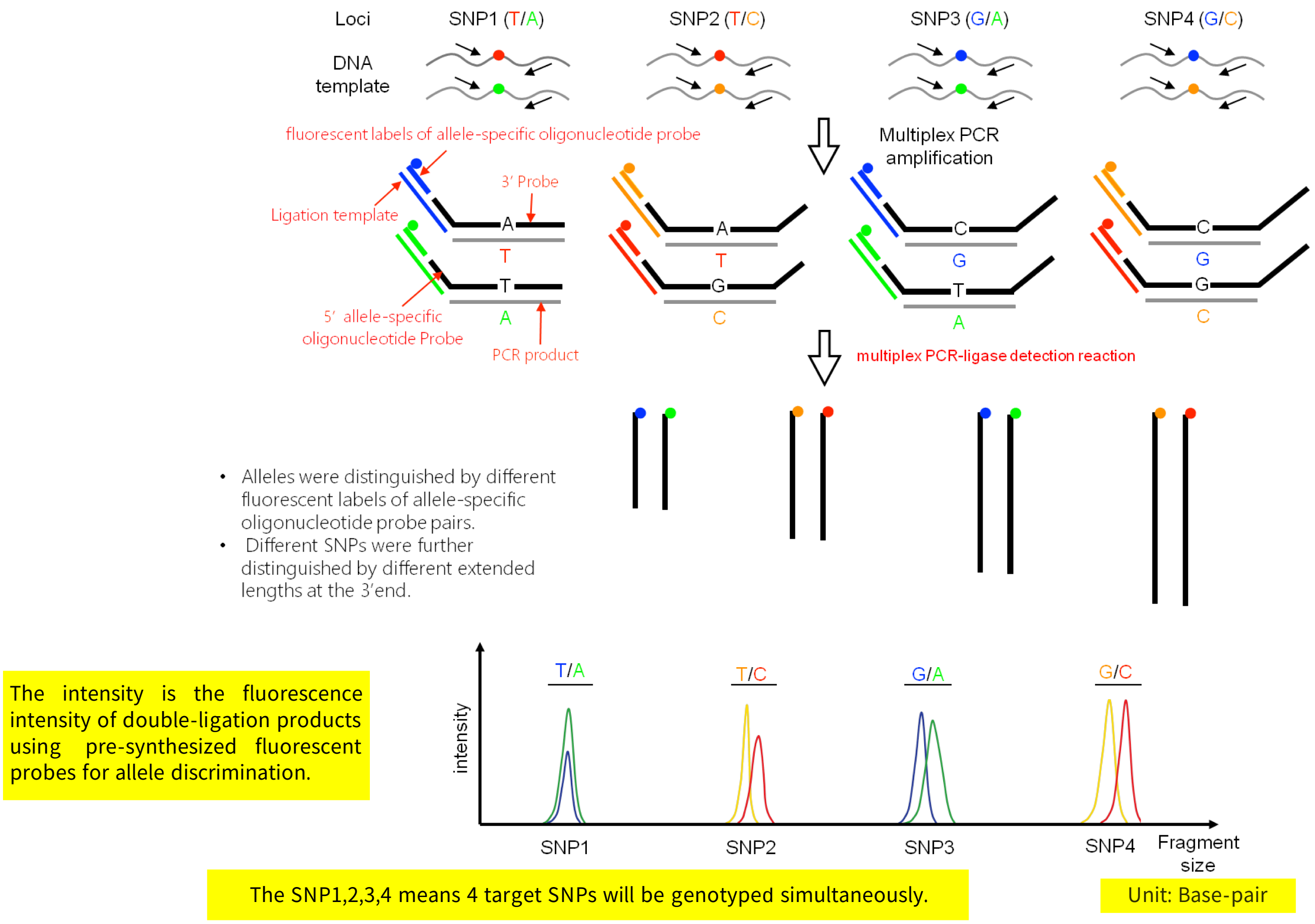

Figure S1.The schematic diagram of iMLDR SNP genotyping.

Sample 110

Note: Each patient in this study corresponds to a sample, the sample 110 was blood sample collected from patient 110 in this study. This figure is presenting the iMLDR genotype results of four SNPs of sample 110. The iMLDR genotype results of all other patients were uploaded as supplementary rawdata which can be opened with GeneMapper 4.1 (AppliedBiosystems, USA) software.

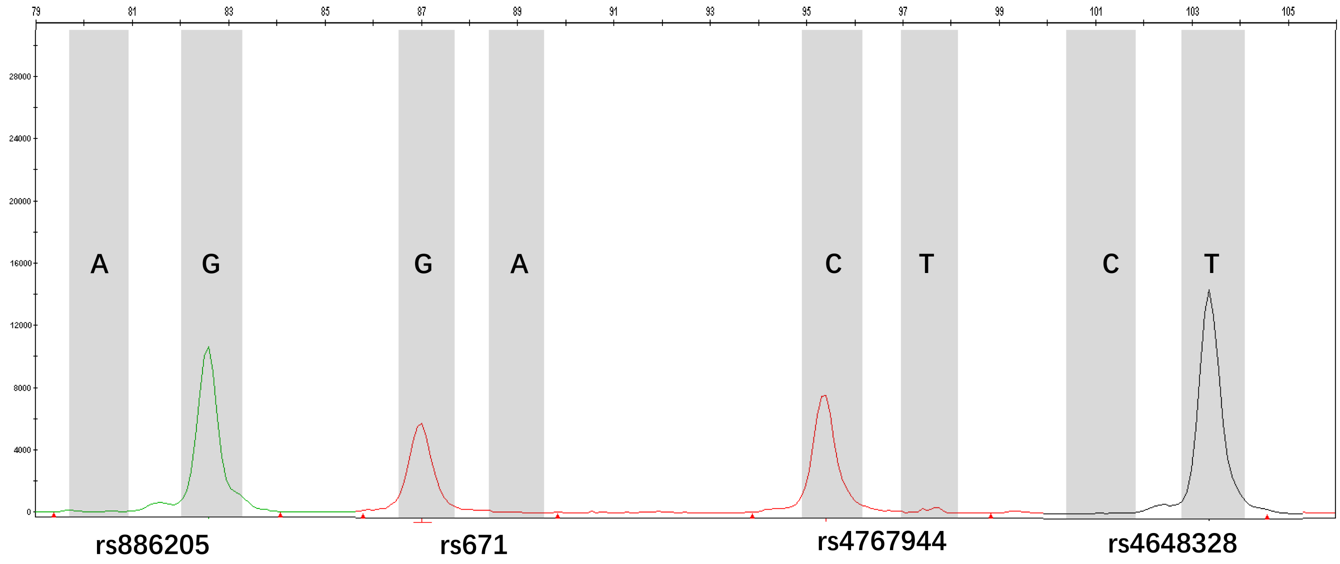

Figure S2.The iMLDR picture of Sample 110.
